# Applying persuasive messages to reduce public outdoor smoking: A pseudo‐randomized controlled trial

**DOI:** 10.1111/aphw.12382

**Published:** 2022-06-29

**Authors:** Sari R. R. Nijssen, Barbara C. N. Müller, Jürgen Gallinat, Simone Kühn

**Affiliations:** ^1^ Environmental Psychology Group, Faculty of Psychology University of Vienna Vienna Austria; ^2^ Behavioural Science Institute Radboud University Nijmegen Netherlands; ^3^ Department of Psychiatry and Psychotherapy University Hospital Hamburg‐Eppendorf Hamburg Germany; ^4^ Lise Meitner Group for Environmental Neuroscience Max Planck Institute for Human Development Berlin Germany

**Keywords:** behavior change, smoking prevention, social influence, persuasive strategies

## Abstract

Despite efforts to create dedicated smoking areas and no‐smoking signs, many smokers continue to light their cigarettes in front of public building entrances—leading to concerns over health consequences for non‐smokers passing by. To increase compliance with no‐smoking requests, behavioral interventions that tap into habitual and automatic processes seem promising. A pseudo‐randomized controlled trial was conducted to assess the differential impact of seven behavioral interventions based on Cialdini's principles of persuasion. Over a period of 9 weeks, the number of smokers was counted (total *n* = 17,930 observations) in front of a German University Medical Center. Relative to a baseline and a control condition, interventions based on the principles of reciprocity, scarcity, and authority were most effective in reducing the number of observed smokers in front of the building entrance (41.5%, 45.7%, and 52.1% reduction rates, respectively). Having observed smokers' behavior in vivo, this study provides substantial evidence for the impact of persuasive strategies on outdoor smoking. In the future, this knowledge should be used to protect non‐smokers from second‐hand smoke by increasing the use of designated smoking areas, leave to another place to smoke, or not smoke at all.

## INTRODUCTION

According to the World Health Organization (WHO), 8 million people die annually due to tobacco. Notably, 1.2 million of those are non‐smokers who are exposed to second‐hand smoke (WHO, 2019). In an attempt to reduce smoking and limit non‐smokers' exposure to tobacco, many countries have adopted indoor smoking bans which prohibit citizens from smoking in public spaces such as hospitality venues, offices, schools, and hospitals (WHO, 2019). While these smoking bans have been very effective in reducing second‐hand smoke exposure (e.g., Verma et al., 2020), they have also inadvertently caused a new problem: Instead of smoking inside, many smokers now cluster outside of public spaces (McGlynn et al., 2018) leading to peak smoke concentrations rivaling (pre‐smoking ban) indoor concentrations (e.g., Klepeis et al., 2007).

Recent data confirm that outdoor smoking can indeed expose non‐smokers to second‐hand smoke, with raised levels of nicotine particles and carbon monoxide found at school entrances (Henderson et al., 2020), hospitality venues (Fu et al., 2016), government buildings (Sureda et al., 2012), and hospitals (Sureda et al., 2010). Even though these levels of second‐smoke are lower than, for example, those in a full‐time smoker's home (Carreras et al., 2019), there is no “safe” level of second‐hand smoke as small concentrations can already have significant health burdens (WHO, 2019). Besides endangering the health of non‐smokers passing by, outdoor smoke particles also permeate adjacent indoor spaces—significantly raising indoor smoke concentrations (Fu et al., 2016; Sureda et al., 2012). This is particularly concerning given that many public spaces, such as schools or hospitals, are meant for people (children and hospital patients) who are especially vulnerable for the effects of second‐hand smoke (Kaufman et al., 2010; Shopik et al., 2012).

To address these issues and reduce the health risk for non‐smokers, two routes to reducing outdoor smoking can be identified: First, the top‐down route involves a legal ban on outdoor smoking. Besides being difficult to enforce, such a ban is politically and ethically sensitive. The second route is more promising: Instead of enforcing a ban, bottom‐up (non‐binding) strategies are directed at the individual smoker and their responsibility for the consequences of their actions. For example, some public spaces such as train stations have been outfitted with designated smoking areas or no‐smoking signs to reduce the risks of second‐hand smoke exposure to non‐smokers (Kaufman et al., 2010).

Unfortunately, until now, these non‐binding strategies appear to be largely ineffective: Many smokers do not comply with no‐smoking requests or dedicated smoking areas and persist smoking in front of building entrances (McGlynn et al., 2018; Navas‐Acien et al., 2016; Russette et al., 2014; Zhou et al., 2016). Thus, the gap between the request and smokers' compliance needs to be bridged. Interventions based on behavioral insights appear suitable for this goal, as they typically sail the narrow strait between top‐down legal obligations, such as a smoking ban, and non‐binding requests, such as a no‐smoking sign. For example, research shows that combining a non‐binding request (e.g., to wash your hands when entering a hospital building) with a persuasive strategy based on behavioral insights often increases compliance (Gaube et al., 2020).

What leads smokers to light up their cigarette, despite no‐smoking requests? A myriad reasons have been identified in the literature: knowledge of the harmful consequences of (second‐hand) smoke, awareness of the no‐smoking request, the extent to which the request is perceived to be enforced or policed, smoking behavior of others in the same setting, own and peer attitudes towards smoking, the perceived convenience of alternative locations such as designated smoking areas, and negative attitudes towards smoking bans all influence a smoker's decision to (not) comply with a no‐smoking request (Lazuras et al., 2009; Russette et al., 2014; Zhou et al., 2016). Specifically in a hospital context, limited physical mobility and fear of straying too far away from hospital grounds appear to be reasons for patients to light up close to the hospital entrance (Shopik et al., 2012).

However, the largest factor guiding smoking decisions is habit and habit strength (Galán et al., 2012; Lacchetti et al., 2001; Lazuras et al., 2009; Li et al., 2010; Parks et al., 2009; Sabidó et al., 2006; Shopik et al., 2012; Zhou et al., 2016). Like many addictive behaviors, smoking is often classified as a habitual behavior (e.g., Stacy & Wiers, 2010), meaning that it is typically repeated frequently, done automatically, and elicited just by being in the environment in which the behavior typically occurs (Orbell & Verplanken, 2010). Research shows that smokers generally score high on measures of habit strength (e.g., Armitage, 2016; Orbell & Verplanken, 2010; Webb et al., 2009). Moreover, the strength of someone's smoking habit has been shown to be predictive of their compliance with smoking bans, both at a behavioral (Orbell & Verplanken, 2010) and self‐report level (Zhou et al., 2016).

Interestingly, despite its automatic character, interventions designed to withhold people from smoking often target more conscious, deliberate decision‐making. Such interventions typically attempt to activate negative cognitions, for example, through warning labels on cigarette packaging (e.g., Mannocci et al., 2012; Müller et al., 2009, 2019). This mismatch between the processing level of the persuasive message and the target behavior could explain the limited impact of such interventions. In contrast, matching health messages to the processing style of the behavior could have potentially positive effects (e.g., Williams‐Piehota et al., 2003). A persuasive strategy that taps into people's automatic responses seems thus more promising to influence peoples smoking behavior in public. Therefore, the present study set out to investigate the possibility of changing smokers' habits with a behavior change approach and persuade them to avoid smoking in front of public buildings.

### Cialdini's principles of persuasion

A useful framework of persuasive strategies is Cialdini's set of principles of persuasion (Cialdini, 2006). These principles have been shown to be effective in a myriad of behavioral change domains, such as healthy eating (Thomas et al., 2017), fostering pro‐environmental behavior (Reese et al., 2014), and compliance with hygiene regulations (de Lange et al., 2012; Gaube et al., 2020). Furthermore, there is abundant evidence for their effectiveness in other compliance domains, such as tax compliance, blood donation requests, and charity donation requests (e.g., Cialdini & Ascani, 1976).

Persuasive messages are likely to be effective in the context of outdoor smoking as well, for two reasons. First, similar to requests to throw trash in the bin in public transport (de Lange et al., 2012) or to disinfect your hands regularly in hospital (Gaube et al., 2020), the request to smoke in designated smoking areas instead of in front of public buildings involves compliance with a descriptive social norm. That is, while most would agree that littering, not washing your hands, and smoking in front of a building are not desired or acceptable behaviors, people still engage in them. Thus, these situations require a change in people's actual observable behavior. Second, similar to other behaviors that have been found to be responsive to persuasive messages, the decision whether or not to light a cigarette is often automatic (e.g., Baxter & Hinson, 2001). Hence, we expect such persuasive messages to be effective in a smoking context as well.

Cialdini identified seven principles of persuasion which can be applied together or in isolation. We will briefly discuss each principle and their relevance to the health domain below.

1. The principle of *reciprocity* is based on the social norm that guides us to respond to a positive action with another positive action (e.g., Molm et al., 2007). This norm to “return the favor” is important in building and maintaining social relationships (Batson, 1998). In the context of health, patients often feel obliged to reciprocate the help and care they receive from nurses by giving them a gift (Morse, 1989). Similarly, patients are more likely to agree to a lifestyle change (e.g., quitting smoking) after their physician does them a small favor (Smith et al., 1986).
2. According to the principle of *scarcity*, we evaluate products or services that appear to be rare or difficult to obtain as more attractive, desirable, and valuable. For example, people are more likely to make healthy food choices when the healthy option is marketed as scarce (Cheung et al., 2015).
3. The *authority* principle holds that those who are in authority positions (e.g., a medical doctor) or otherwise have great influence (e.g., social media influencers) have more credibility in the eyes of the recipient. As a consequence, people are more likely to comply with requests of an authority (Dolinksi et al., 2020). For example, research shows that people are more likely to comply with recommendations for a healthier diet when it is supported by a dietician (Thomas et al., 2017).
4. *Commitment* and *consistency* refer to our tendency to be consistent in our actions. That is, we like to act in accordance with our core beliefs and past actions. Therefore, if we are reminded on any of our past actions or beliefs, we tend to subsequently act similarly in the present moment (e.g., Baca‐Motes et al., 2013). In the context of smoking cessation, smokers who have committed to quitting smoking by signing a contract or setting up a dedicated bank account for their extra savings are more likely to be successful in their quitting attempt (e.g., Giné et al., 2010).
5. *Social proof* refers to the strong influence others have on our behavior (Nolan et al., 2008). If many people perform a certain behavior or have a certain opinion, we are likely to adopt the same behaviors and opinions. For example, visitors and patients in a hospital were more likely to use hand sanitizer when seeing others perform the behavior at the same time (Gaube et al., 2020).
6. The *liking* principle entails that we are more likely to comply with requests of those we like; we like those who are similar to us, who compliment us, or who work with us towards the same goal. In the health domain, it has been demonstrated that physicians consistently give more diagnostic and health information to patients who are more similar to them (Verlinde et al., 2012).
7. Lastly, the principle of *unity* overlaps with principles 5 and 6 in the sense that unity also taps into our fundamental need to belong (Baumeister & Leary, 1995). Essentially, the principle of unity entails that we are more likely to comply with requests regarding the group we (want to) belong to. For example, when a health professional emphasizes shared goals between themselves and the patient, the patient is more likely to adopt and maintain a healthy lifestyle (Johnson, 2007).

### The current study

Designated smoking areas help to reduce the risks of second‐hand smoke exposure to non‐smokers (e.g., Kaufman et al., 2010), but overall, compliance rates are rather low (McGlynn et al., 2018; Navas‐Acien et al., 2016; Russette et al., 2014; Zhou et al., 2016). To increase compliance, the goal of the present study was to gather empirical evidence for effective ways of enforcing the smoke‐free policy in front of the main entrance of hospitals. To reach that goal, we translated Cialdini's seven principles of persuasion into speaker messages. These were broadcast at the entrance of a public building (a hospital), and the number of smokers at the entrance and the designated smoking‐area was measured. We hypothesize that persuasive messages based on Cialdini's principles would be (1) effective in reducing the number of smokers in front of the hospital entrance and (2) successfully increase compliance with the request to smoke in designated smoking areas instead. No specific hypotheses were formulated regarding the relative impact of each individual principle.

## METHODS

The field study consisted of a pseudo‐randomized controlled trial with seven treatment conditions and one control condition. The study was conducted over a period of 9 weeks in August and September 2020 at a large university medical center (±1,500 beds, >500.000 patients per year, 13.560 employees) in northern Germany. While data collection was conducted with human participants, no personal identifying information was collected, and thus, no informed consent was obtained. The experimental procedure was approved by the local ethics committee.

### Procedure

A visual overview of the study timeline can be found in Table 1. Baseline measurements were taken in week 1, followed by the intervention period in weeks 2–9. Measurements were taken on all days of the week (Monday to Sunday). In the intervention period, one intervention condition (seven treatment and one control) was implemented per day. The eight different conditions were assigned to days of the week in a counterbalanced order, so that each condition was implemented once on each day of the week. On each day, the number of smokers was observed in 15‐min intervals between 9:00 AM and 5:00 PM.

**TABLE 1:** An overview of the study timeline with 1 week of baseline measurements and 8 weeks of intervention measurements

Week	Monday	Tuesday	Wednesday	Thursday	Friday	Saturday	Sunday
1	Baseline	Baseline	Baseline	Baseline	Baseline	Baseline	Baseline
2	T1	T2	T3	T4	T5	T6	Control
3	T2	T3	T4	T5	T6	Control	T7
4	T3	T4	T5	T6	Control	T7	T1
5	T4	T5	T6	Control	T7	T1	T2
6	T5	T6	Control	T7	T1	T2	T3
7	T6	Control	T7	T1	T2	T3	T4
8	T7	T1	T2	T3	T4	T5	T6
9	Control	T7	T1	T2	T3	T4	T5

*Note*: The overview additionally shows the specific counterbalanced assignment of the various conditions (control and treatments T1–T7) to days of the week.

### Intervention

For each condition, an audio message was recorded by a professional voice actress. Messages were recorded in German. The intervention messages were matched for length, tone of voice, speed, and affective tone. On intervention days, the audio message of the respective treatment or control condition was played on repeat every 5 min via a speaker system at the entrance of the hospital. During the baseline period, no audio messages were played. The audio messages were based on Cialdini's principles of persuasion reciprocity, scarcity, authority, commitment and consistency, social proof, liking, and unity (Cialdini, 2006). The content of each intervention message is presented below in translated form. Original audio files and message texts are available on the Open Science Framework.^1^

#### Reciprocity

For the reciprocity treatment condition, we constructed a message tapping into people's tendency to reciprocate by stating that the hospital took the effort of setting up special smoking areas for smokers and inviting them to return the favor by making use of it. The full message script was “Dear smokers, [name of hospital] has set up special smoking areas for you in order to offer all smokers a comfortable and dry place to smoke. You can return this favor and take advantage of these special smoking areas. Thanks for your support.”

#### Scarcity

The principle of scarcity was applied to the current study by informing smokers that the possibilities to smoke on the hospital campus are limited, and the designated smoking areas are a rare location where smoking is still allowed. The full message script was “Dear smokers, smoking is actually forbidden throughout the [name of hospital]. The possibilities to still smoke are very limited; your last chance to smoke is in the designated smoking areas. Thanks for your support.”

#### Authority

We applied this principle by emphasizing the authority of the hospital director in the request to avoid smoking in front of the hospital entrance. The full message script was “Dear smokers, to protect our patients, the Medical Director has banned smoking in all buildings and entrance areas. He asks you to use the existing smoking areas instead. Thanks for your support.”

#### Commitment and consistency

In relation to the current study, a common belief people adhere to is that our health is valuable and that certain groups are vulnerable. For the current study, we thus appealed to the protection of patient; once recipients of the audio message would have agreed with the first part of the message, the likelihood they would likewise agree with the second part would increase. The full‐text of the message script was “Dear smokers, do not you also want the best for our [name of hospital] patients? Then use the designated smoking areas and help all patients to recover as quickly as possible. Thanks for your support.”

#### Social proof

To increase the sense that many smokers avoid smoking in front of the hospital entrance, the audio message was constructed as follows: “Dear smokers, almost 75% of smokers use the designated smoking areas. Together with your fellow smokers, you can help to support and expand this majority by using the designated smoking areas. Thanks for your support.”

#### Liking

To increase smokers' liking of the hospital by means of similarity/similar goals and values, we constructed the following message: “Dear smokers, the promotion of health is our goal, and certainly yours, too. In order to achieve our common goal, please use our designated smoking areas if you would like to smoke. Thanks for your support.”

#### Unity

In this study, we aimed to increase unity between smokers and the hospital it was referred to a shared identity between the two by stating “Dear smokers, we are all part of the [name of hospital] community. Together we can make the [name of hospital] a place where everyone feels comfortable. You can help by making use of the designated smoking areas. Thanks for your support.”

#### Control

To control for potential confounding effects of the presence of an audio message on people's decision to smoke or not to smoke in front of the hospital entrance, a control message was constructed. The full message script was “Dear patients, visitors and employees, we look forward to welcoming you to the [name of hospital]. A place where you will be helped. We are happy to help you. Contact us.”

### Measurements

Eighteen trained observers recorded the number of smokers in two locations: (1) in front of the entrance to the main building (location size: 472.35 m^2^) and (2) at the designated smoking area approximately 100 m away from the entrance (location size: 110.91 m^2^). Measurements were taken at 15‐min intervals for the duration of the intervention timeline, resulting in 33 measurements per day. Observers were instructed by an expert observer along specific guidelines for classifying a person in the observation areas as smoking.^2^ To be classified as smoking, people had to either be rolling a cigarette, lighting a cigarette, smoking a cigarette or e‐cigarette, or about to put out a cigarette. Observers were instructed to note the number of smokers as inconspicuously as possible by using their phone to record their observations, and to avoid counting children and passers‐by. Observers were blind to the study's hypothesis. Furthermore, they were randomly assigned to observation timeslots.

## RESULTS

### Descriptives

Throughout the intervention period, in total, *N* = 17,930 observations were made. Of those, *N* = 6,759 observations were classified as people smoking. It should be noted that relatively more observations of smokers were made in the dedicated smoking areas (*N* = 5,295) than in front of the hospital entrance (*N* = 1,464). Per measurement period, our observers recorded on average *N* = 3.27 smokers: *N* = 2.56 in the dedicated smoking areas and *N* = 0.71 at the hospital entrance. Since the two dependent measures (smokers in front of the hospital entrance and smokers in the dedicated smoking areas) were not correlated (*r* = .03, *p* = .221), two univariate analyses of variance (ANOVAs) were conducted to assess the impact of our intervention.^3^

### Main analyses

#### Number of smoking observations at the hospital entrance

**FIGURE 1:**
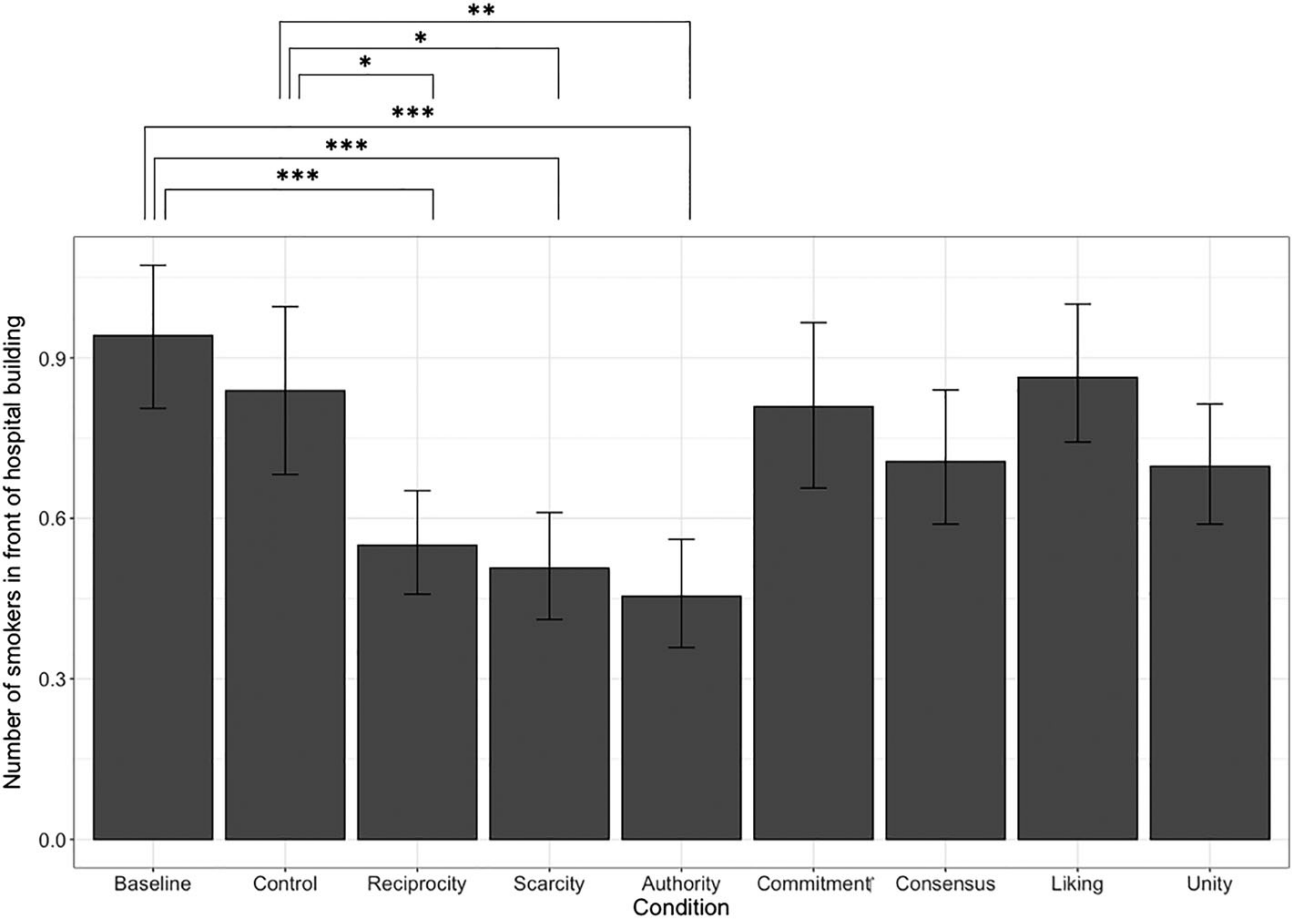
The number of people in front of the hospital building who were classified as “smoking” per condition. *Note*: *p < .050, **p < .010, ***p < .001

The assumption of normality was met but Levene's test indicated inequality of variance (*F*[8, 2,059] = 5.53, *p* < .001). Hence, to analyze the impact of the Persuasion type on the mean number of observed smokers at the hospital entrance, a Welch ANOVA was conducted with Games‐Howell post hoc tests. Results show that persuasion type had a significant effect on the number of observed smokers, *F*_*Welch*_ (8, 2059) = 8.12, *p* < .001, ω^2^ = 0.03. Post hoc tests revealed that this effect was driven by the persuasion types reciprocity (*p*_*baseline*_ < .001, *p*_*control*_ = .046, reduction rate 41.5%), scarcity (*p*_*baseline*_ < .001, *p*_*control*_ = .012, reduction rate 45.7%), and authority (*p*_*baseline*_ < .001, *p*_*control*_ = .002, reduction rate 52.1%). In those three conditions, significantly fewer people were classified as smokers than in the control or baseline conditions. The remaining Persuasion types of commitment, social proof, liking, and unity had no significant effect on the number of smokers in front of the hospital entrance relative to baseline (all *p*'s > .217) or control (all *p*'s > .886). Results for the hospital entrance are summarized in Figure 1.

#### Number of smokers in the designated smoking areas

**FIGURE 2:**
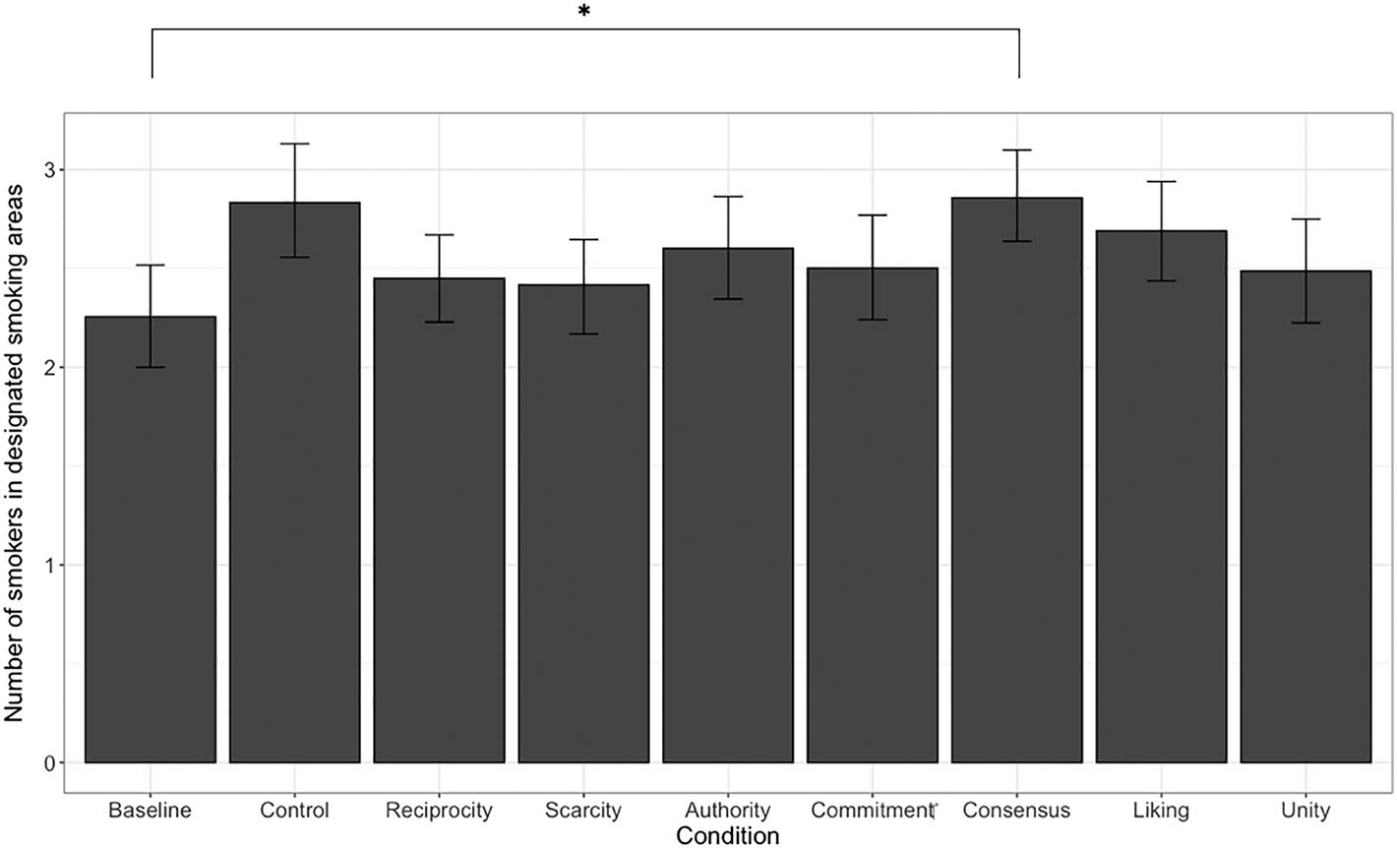
The number of people in the designated smoking areas who were classified as “smoking” per condition. *Note*: *p < .050, **p < .010, ***p < .001

The assumption of normality was met but Levene's test indicated inequality of variance (*F*[8, 2,058] = 2.14, *p* = .030). Hence, to analyze the impact of the Persuasion type of the mean number of observed smokers at the designated smoking areas, a Welch ANOVA was conducted with Games‐Howell post hoc tests. Again, Persuasion type had a significant effect on the number of observed smokers, *F*(8, 2058) = 2.41, *p* = .014, ω^2^ = 0.01. The result of post hoc tests indicated that only the Social Proof persuasion strategy significantly increased the number of smokers in the designated smoking areas relative to the baseline condition (*p* = .021) but not the control condition (*p* = 1.000). All other persuasion types had no effect on the number of smokers relative to baseline (all *p*'s > .311) or control (all *p*'s > .372). Results for the designated smoking areas are summarized in Figure 2.

## DISCUSSION

The current study aimed to investigate different messages to reduce the number of smokers in front of public buildings by gathering empirical evidence for the effect of various types of persuasive messages. A pseudo‐randomized controlled trial was conducted with one control condition and seven treatment conditions, which consisted of Cialdini's seven principles reciprocity, scarcity, authority, commitment, social proof, liking, and unity. Results provide clear evidence for the effect of persuasive interventions on the number of people observed smoking in front of the hospital entrance, as well as at the designated smoking areas. Specifically, the authority, reciprocity, and scarcity persuasive interventions significantly reduced the number of people observed smoking in front of the hospital entrance with relative to our baseline condition. Specifically, the authority intervention reduced the number of observed smokers with 52.1%, the scarcity intervention by 45.7%, and the reciprocity intervention 41.5%. At the designated smoking areas, the social proof persuasive intervention was found to significantly increase the number of observed smokers with 27.1%. Importantly, the design of the current study allowed for a direct comparison of various persuasive interventions. Our results thus provide convincing evidence for the relative impact of authority, reciprocity, scarcity, and social proof‐based persuasive interventions on smoking behavior, in contrast to other persuasive strategies. In the subsequent, we will further interpret these findings in the study context. We will also discuss advantages and limitations of field research and the current study design. Lastly, we will present recommendations for the implementation of the current findings (Figure 2).

The most effective persuasive message in the current study was based on Cialdini's principle of authority: messages that highlighted that the hospital director request to avoid smoking in front of the hospital entrance lead to fewer smokers in this area compared to the two control conditions. In any behavioral intervention, the effectiveness of the persuasive strategy depends not only on the fit between the intervention and the psychological determinants of the behavior but also on the setting in which the intervention and the to‐be‐changed behavior take place (Fishbein & Cappella, 2006). Indeed, many studies have shown cultural differences in sensitivity to Cialdini's principles of persuasion (Wosinka et al., 2001). For example, people from individualistic cultures are typically more sensitive to messages about scarcity and consistency, while people from collectivistic cultures perceive authority, reciprocity, social proof, and liking as more persuasive (Orji, 2016; Petrova et al., 2007; Wosinka et al., 2001).

The current study took place in a hospital setting in northern Germany. Generally speaking, Germany scores relatively high on cultural values expressing the importance of authority such as Hofstede's power distance (Hofstede, 1984) and Schwartz's hierarchy (Schwartz, 1992)—especially compared to other Western countries such as Denmark or Canada (Goeveia & Ros, 2000). The hospital setting of the study may have further induced this sense of authority. Classically, the medical doctor is seen as an authority figure and patients typically comply with their treatment recommendations (Frosch et al., 2012; Schmieder et al., 2019). Altogether, the effectiveness of the authority message in the current study is best understood when taking into consideration the setting of the study: in a country with relatively high power distance and hierarchy, in a setting that directly primes authority. When considering implementing an authority‐based persuasion strategy, researchers and practitioners should thus consider the cultural context in which they operate: A context in which values such as power distance and hierarchy are dominant are perhaps better suited for authority messaging than situations in which, for example, anti‐authority sentiments prevail (e.g., Murphy et al., 2021).

In addition to authority, the reciprocity and scarcity persuasive interventions were effective in reducing the number of observed smokers in front of the hospital as well. Similar to the effect of authority, the findings regarding reciprocity can probably also be explained by the hospital context of the study. Most (if not all) potential smokers in this study were in some way related to the hospital: They may have been receiving treatment from the hospital (patients), their loved ones might have been treated there (visitors), or they may be getting a monthly paycheck from the hospital (staff). In this sense, the hospital is providing them with a service—and a message reminding potential smokers they can return the favor by lighting their cigarette somewhere else might tap into feelings of obligation to return this favor (see for similar arguments, Morse, 1989; Verlinde et al., 2012). Regarding scarcity, the interpretation of the obtained effects could be sought in smokers' perception of dedicated smoking areas. Research confirms that smokers appreciate the presence of a designated smoking location in a no‐smoking environment such as a university or hospital campus, as it is more convenient to walk to a designated smoking area than having to travel all the way off campus (e.g., Shopik et al., 2012; Zhou et al., 2016). From this perspective, tapping into scarcity makes sense: The scarcity message emphasizes that there it at least a limited opportunity for them to use a conveniently located space, which is better than not being allowed to smoke on the hospital campus at all.

An interesting apparent paradox can be noted in our pattern of results. While the authority, scarcity, and reciprocity messages were effective at reducing the number of smokers in front of the hospital entrance, it was only the social proof message that significantly increased the number of smokers in the designated smoking areas. People typically prefer to perform behaviors that are in line with the rest of the group (Nolan et al., 2008). That is, people do not like to stand out from the crowd. Furthermore, smoking is specifically associated with socialization (Sureda et al., 2015). Thus, it makes sense that the social proof message was efficient in persuading smokers to use the designated smoking areas: When they feel that all their peers are doing the same, they are more inclined to copy the behavior.

Importantly, from the measurements obtained in front of the hospital entrance, we can conclude that the reciprocity, authority, and scarcity messages induced people to reconsider lighting their cigarette in that location—but we cannot be certain whether they decided not to smoke at all, or smoke later in a different location other than the dedicated smoking area. Longer observations that focus on the subsequent behavior of the smokers should be implemented in future research to understand this discrepancy. Yet, from the results obtained in the dedicated smoking areas, we can conclude that the social proof message was actually effective at persuading people to smoke in a different location rather than in front of the hospital. Indeed, people could not hear the audio messages from the designated smoking areas; the effect of social proof on the number of observed smokers thus only reflects those who actually heard the message at the main entrance and complied with the request.

All behavioral outcomes (not smoking at all, smoking in a different location, or smoking specifically in the designated areas) limit non‐smokers' exposure to cigarette smoke and are therefore positive. Yet the divergent pattern of results is psychologically interesting, as it can perhaps be best understood by considering the motivational mechanisms behind the different behavioral outcomes. Deciding not to smoke in that specific location involves a passive choice (inhibiting an action), while deciding to smoke in a specific area involves a more active choice (choosing an alternative action; e.g., Boecker et al., 2013; Verbruggen & Logan, 2008). One could imagine the authority message being particularly persuasive at convincing people not to smoke in that moment, while the message informing smokers that most other smokers use the designated smoking areas is more motivating to actively decide to pursue a different course of action. Of course, this is a post hoc interpretation that should be corroborated in a more tightly controlled lab setting to test potential differences between the different persuasive strategies in their impact on different behavioral outcomes.

Some limitations to the current research can be noted as well. First, the exposure period of the intervention was limited. The intervention period lasted for 2 months, during which the different persuasive messages were played in a counterbalanced fashion. In this setting, we found clear impact on smoking behavior of the authority, reciprocity, and scarcity messages. However, it is difficult to estimate whether this impact is maintained when the messages are played over a longer period of time or when only one or two messages are played. The repetition of a single message may indeed lead to habituation and any behavioral change may fade out over time (e.g., Allcott & Rogers, 2014; Halpern & Sanders, 2016; Taubinsky, 2013). To investigate this, follow‐up research is needed that looks into the longer‐term impact of these persuasive messages on smoking behavior.

A second limitation is our lack of demographic information. Since this was an in vivo observational study, no per‐participant demographic information is available. Yet, since the study was conducted at a university medical center in a large city in northern Germany, we assume the sample is representative of the local population. In addition, we cannot distinguish in our data between hospital patients, visitors, or hospital staff. Perhaps, the various persuasive messages had different effects on these subpopulations. For example, our interventions were based on the theoretical assumption that smoking behavior is typically an automatic behavior, cued by the environment (Orbell & Verplanken, 2010). Nonetheless, it has been established that novice smokers have less strongly entrenched habits and use more attentional resources when smoking, compared to experienced smokers or people who smoke more frequently (Baxter & Hinson, 2001; Field et al., 2006). However, since we did not measure the habit strength of the observed smokers in this study, we cannot conclude anything about the different interventions and how they were effective at different levels of habit strength. Ways to address this caveat could be to collect qualitative data or conduct a lab study in future research. Yet one could also argue that an intervention in real life involves different populations at the same time anyway. Thus, the fact that our data show that the intervention works on average across all subpopulations is perhaps sufficient to recommend its implementation in practice.

A third limitation is potential environmental factors that could explain our results. Yet given that the intervention (including baseline measures, the control condition, and all treatment conditions) was carried out at exactly the same location, and that the pseudo‐randomized study design (see Table 1) was set up in such a way that all conditions were displayed on each day of the week, we deem it unlikely that the significant differences we found between conditions result from environmental factors.

The strength of this research lies in our ability to draw conclusions about the relative impact of different behavioral change interventions. In typical behavior change research, an intervention is compared to a control group. For example, guests in a hotel who were exposed to a social proof intervention were more likely to reuse their towels during their stay than guests who did not receive such an intervention (Reese et al., 2014). However, our psychological landscape is much broader than social sensitivity alone. Yet, because of the research design, the effect of social proof cannot be compared to other interventions, and therefore, no conclusions can be drawn about the relative impact of other interventions on people's behavior. This is a shortcoming because much more effective intervention strategies may be missed, with important consequences for the implementation of interventions in the field. In the current research, we therefore contrasted seven types of persuasive factors in a counterbalanced fashion. From the results, we can draw clear conclusions about the relative impact of all these factors—showing that authority, reciprocity, and scarcity were effective in reducing the number of observed smokers relative to commitment, liking, unity, and social proof. The effect of these persuasive strategies did not differ significantly from one another, so their implementation should have similar effects. In persuading smokers to make use of designated smoking areas, social proof was the only effective persuasive strategy—thus paving a clear path for practitioners in government, business, or public administration wishing to increase the use of dedicated smoking areas in a no‐smoking environment.

The current study yields three specific recommendations for behavioral interventions aimed at reducing the number of outdoor smokers. First, in front of public buildings that are typically associated with authority (e.g., a hospital, city hall, and a police station), persuasive audio messages tapping into smokers' sense of authority may be helpful in convincing smokers not to smoke in that specific location. Second, generally speaking, persuasive audio messages tapping into our sense of scarcity or reciprocity may be helpful in persuading smokers to not smoke there. Third, when you are trying to convince smokers to make use of designated smoking areas, tapping into their sense that it is the norm to smoke in these areas may prove fruitful.

Altogether, this observational field study is the first of its kind to investigate the impact of persuasive strategies on outdoor smoking. By observing real behavior, we were able to show that persuasive strategies can be effective in reducing the number of smokers in front of a hospital entrance. In the future, this knowledge can be used to protect non‐smokers from second hand smoke by increasing compliance rates in smokers to use designated smoking areas, leave to another place to smoke, or to not smoke at all.

## CONFLICT OF INTEREST

No potential competing interest was reported by the authors.

## ETHICS STATEMENT

The experimental procedure was approved by the local ethics committee.

## Data Availability

Data is stored on the Open Science Framework [https://osf.io/qfxy6/].

## ENDNOTES

^1^
All materials are available on the Open Science Framework (https://osf.io/qfxy6/).

^2^
Original instructions are accessible on the Open Science Framework (https://osf.io/qfxy6/).

^3^
Data are stored on the Open Science Framework (https://osf.io/qfxy6/).
